# Determinants of anemia among women and children in Nepal and Pakistan: An analysis of recent national survey data

**DOI:** 10.1111/mcn.12478

**Published:** 2017-08-31

**Authors:** Kassandra L. Harding, Víctor M. Aguayo, Grace Namirembe, Patrick Webb

**Affiliations:** ^1^ Friedman School of Nutrition Science and Policy Tufts University Boston Massachusetts USA; ^2^ United Nations Children's Fund (UNICEF) Regional Office for South Asia Kathmandu Nepal

**Keywords:** anemia, children, Nepal, Pakistan, South Asia, women

## Abstract

Anemia remains one of the most intractable public health challenges in South Asia. This paper analyzes individual‐level and household‐level determinants of anemia among children and women in Nepal and Pakistan. Applying multivariate modified Poisson models to recent national survey data, we find that the prevalence of anemia was significantly higher among women from the poorest households in Pakistan (adjusted prevalence ratio [95% CI]: 1.10 [1.04–1.17]), women lacking sanitation facilities in Nepal (1.22 [1.12–1.33]), and among undernourished women (BMI < 18.5 kg/m^2^) in both countries (Nepal: 1.10 [1.00–1.21] and Pakistan: 1.07 [1.02–1.13]). Similarly, children in both countries were more likely to be anemic if stunted (Nepal: 1.19 [1.09–1.30] and Pakistan: 1.10 [1.07–1.14]) and having an anemic mother (Nepal: 1.31 [1.20–1.42] and Pakistan: 1.21 [1.17–1.26]). Policies and programs need to target vulnerable and hard‐to‐reach subpopulations who continue to bear a disproportionate burden of anemia. Covariates of poverty underpin rates of anemia among children and their mothers, but income growth alone will not suffice to resolve such deeply entrenched problems. Greater understanding of the relative role of various diet, health, sanitation, and educational factors by local context should guide investments to resolve anemia in tandem with stunting and maternal underweight.

## 1 INTRODUCTION

Anemia remains a major public health problem, affecting one third of all adults and almost two billion people worldwide (Global Burden of Disease DALYs et al., 2015; Kassebaum et al., 2014). Defined broadly as a condition associated with lower than normal hemoglobin concentration, anemia impairs the circulation of oxygen in the blood, which in turn has detrimental effects on maternal and birth outcomes, suboptimal child growth, impaired learning, and reduced work productivity and income earning during adulthood (International Food Policy Research Institute, 2016). Such effects compounded across the entire populations lead to significant economic losses, through foregone gross domestic product (GDP) and treatment costs (Balarajan, Ramakrishnan, Özaltin, Shankar, & Subramanian, 2011).

The causes of anemia in low‐income and middle‐income countries are typically grouped into three broad categories: nutritional deficiencies, infectious diseases, and genetic hemoglobin disorders (Warrell, Cox, Firth, & Benz, 2003). Iron deficiency, which may account for more than 60% of all cases of anemia (Global Burden of Disease DALYs et al., 2015), was associated with roughly 120,000 preventable deaths in 2010 (two thirds of them among women; Lim et al., 2012) and more than 5% of all years lost to disability (YLD) globally (2013).

In 2012, South Asia accounted for 38% of the entire world's YLD associated with anemia (Kassebaum et al., 2014; Stevens et al., 2013). The region has made little progress in resolving this problem, despite decades of economic growth. The modeled data suggest that between 1995 and 2011, the prevalence of anemia among nonpregnant women in South Asia declined slightly from 53% (95% credibility interval: 42–64%) to 47% (33–59%) and there was almost no change among pregnant women between 1995 (53% [43–63%]) and 2001 (52% [40–63%]; Stevens et al., 2013). Change in anemia among children under 5 years of age was more noticeable, falling from an estimated 70% (59–78%) to 58% (44–69%) during the same period (Stevens et al., 2013). Trends in the same modeled data suggest a rising prevalence among children in Afghanistan, Pakistan, and Sri Lanka between the early 2000s and 2012, with a similar rise among nonpregnant women in Afghanistan and Pakistan (Stevens et al., 2013). In other words, anemia has persisted across South Asia and remains a moderate (20.0–39.9%) or a severe (≥40%) public health problem among children and nonpregnant women in most of the region (UNICEF et al., 2001).

These levels of anemia pose a significant challenge to policymakers tasked with meeting global targets for anemia reduction in line with the World Health Assembly's goal of reducing the prevalence among women of reproductive age (WRA) globally by 40% by 2025 (WHO, 2014). To achieve such targets, a better understanding is needed of the many factors that contribute to anemia in different parts of South Asia, as well as good empirical evidence of successful interventions (International Food Policy Research Institute, 2015; Raykar, Majumder, Laxminarayan, & Menon, 2015). For example, trends in and determinants of anemia among women have been recently examined in relation to Bangladesh and India (Balarajan, Fawzi, & Subramanian, 2013; Kamruzzaman, Rabbani, Saw, Sayem, & Hossain, 2015). However, such an analysis has not yet been conducted for Nepal or Pakistan, despite the availability of relatively recent survey data. Thus, the main aim of this study was to assess recent trends and determinants of anemia among women and young children in Nepal and Pakistan in order to generate strong evidence on outcome patterns and their drivers that can support policymakers in the region to identify and implement better evidence‐based interventions.

**Key messages**

- Anemia was prevalent among children and women in our sample from Nepal and Pakistan, indicating a moderate to severe public health concern in these populations.
- Stunted children ‐ over 40% both countries – were more likely to be anemic compared with those not stunted, and having an anemic mother was associated with a higher odds of anemia among children. Among women, being thin increased the likelihood of anemia in both countries. Given the overlaps of anemia, stunting, and thinness, there are opportunities to target multiple conditions in the same individuals and households simultaneously.
- South Asia includes countries such as Bangladesh and Nepal that have strong policies to address anemia, while other countries in the region that suffer from a higher prevalence of anemia could benefit from a stronger policy and programme commitment to targeting anemia.

## 2 METHODS

### 2.1 Data sources

The Nepal Demographic and Health Survey (DHS) 2011 and Pakistan's National Nutritional Survey (NNS) 2011 datasets were obtained. These surveys are the most recent nationally representative cross‐sectional surveys documenting the nutrition situation of women and children in Nepal and Pakistan. Nepal DHS 2011 used a two‐stage stratified cluster sample designed to provide representative estimates for urban and rural areas, and the three ecological zones cross‐classified with five development regions (i.e., “domains”). The survey used the enumeration areas (EAs) from the 2001 Nepal census. EAs were the primary sampling unit (PSU) and were selected from each domain with probability proportional to size. Households within EAs were selected using a systematic sampling technique with a random selection within clusters. A total of 13,485 WRA (15–49 years old) were expected to complete the survey.

Pakistan's NNS 2011 also used a multistage cluster sampling methodology to produce representative data at the national and provincial levels as well as urban and rural. The survey was conducted in the four provinces and three additional regions: Azad Jammu and Kashmir, Gilgit Baltistan, and Federally Administered Tribal Areas. The Federal Bureau of Statistics's sampling frame and enumeration blocks were used. These enumeration blocks were the PSU, and households were sampled within the selected PSUs. In total, 30,000 households were expected to be sampled and 9,836 WRA for biochemical analyses. More details on the sampling and implementation of these surveys have been published in the respective final reports (Aga Khan University, 2011; Ministry of Health and Population et al., 2012; Ministry of Public Health et al., 2014).

### 2.2 Analytic samples

WRA were included in this analysis if they (a) had data on hemoglobin concentration or data on presence of anemia, (b) had complete data on household‐level and individual‐level modules, and (c) were not pregnant. A total of 5,794 WRA were included in the analysis for Nepal and 8,324 in the analysis for Pakistan.

Children <5 years old were included in the analysis if they (a) had data on hemoglobin concentration or data on presence of anemia and (b) had complete data on household‐level and individual‐level modules. In total, 2,088 children from Nepal and 8,968 children from Pakistan were included in the respective analyses.

### 2.3 Anemia

Anemia was defined as hemoglobin concentration <120 g/L for WRA and <110 g/L for children <5 years old (UNICEF et al., 2001). Hemoglobin status was evaluated using capillary blood and the HemoCue rapid testing in Nepal and evaluated using venous blood in Pakistan. Hemoglobin concentration was adjusted for altitude in Nepal, but not Pakistan.

### 2.4 Predictors of anemia

We conducted a literature review of predictors of anemia in South Asia and tested factors that have been considered previously, with emphasis on two recent studies that explored the correlates of anemia using India's DHS 2005–2006 and Bangladesh's DHS 2011 (Balarajan et al., 2013; Kamruzzaman et al., 2015). Improving the comparability of analyses across country studies in the region is an important goal underpinning the work reported here. The factors tested in Nepal and Pakistan differed slightly based on the availability of data. For example, the Pakistan NNS included dietary and micronutrient status data, whereas the Nepal DHS did not.

A variable for wealth index was created using principal component analysis. This derived variable was already included in the Nepal dataset and was created by us using the Pakistan dataset, using the method to create this variable from the final report for the Pakistan NNS. Food security scores were calculated using methods used in Pakistan's NNS (Aga Khan University, 2011).

The 2006 World Health Organization growth standards were used to calculate children's height‐for‐age, weight‐for‐age, weight‐for‐height, and body mass index (BMI)‐for‐age *z*‐scores and define stunting, wasting, underweight, and overweight, respectively. Variables for these *z*‐scores existed in the Nepal dataset and were calculated in the Pakistan dataset using the zscore06 in Stata (Leroy, 2011).

Serum retinol, serum ferritin, plasma zinc, vitamin D, and calcium were evaluated in Pakistan to determine vitamin A deficiency, low ferritin, zinc, vitamin D, and calcium deficiency. Alpha 1 acid glycoprotein was used to adjust serum retinol, serum ferritin, and plasma zinc for inflammation (Engle‐Stone et al., 2011; Thurnham et al., 2010).

### 2.5 Statistical analysis

The prevalence of anemia among women and children was calculated for Nepal using reported sample weights. After log binomial models failed to converge, modified Poisson models were used to evaluate factors associated with anemia and estimate prevalence ratios, adjusting for the clustered design of the surveys. This approach was chosen because the outcome was not rare (>10%), and therefore, log binomial and modified Poisson models were more appropriate than logistic regression (Balarajan et al., 2013; Barros & Hirakata, 2003; Deddens & Petersen, 2008; Zou, 2004). Factors that were extremely rare or common (<5% or >95%) were not included in the model, and backwards stepwise processes were used to identify parsimonious models to explain predictors of anemia (all *p* < .05) in each country for each group of WRA and children <5 years old.

## 3 RESULTS

The characteristics of the study populations included in this analysis are described in Table 1. Children in Nepal and Pakistan were on average (mean [SD]: 32.4 [15.5] and 24.9 [16.2]) months old. In Nepal, 43% of children were stunted, 11% wasted, and 30.0% underweight. In Pakistan, 42% of children were stunted, 16% wasted, and 32% underweight.

**Table 1:** Selected characteristics of the study populations^a^

	Nepal (weighted)^b^	Nepal (unweighted)	Pakistan
Mothers	*n =* 5,794	*n =* 5,794	*n =* 8,324
Rural	86.2	71.7	59.2
Improved water source	91.6	91.0	95.7
Number of household members (*n*)	5.6 (2.7)	5.6 (2.7)	7.3 (3.1)
Age (years)	29.1 (9.8)	29.0 (9.8)	30.8 (6.2)
Number of births (*n*)	2.0 (1.7)	2.0 (1.7)	3.9 (2.0)
Body mass index (kg/m^2^)	21.4 (3.4)	21.5 (3.4)	23.2 (5.1)
Literate	67.7	69.6	44.9
Anemic	34.4	31.7	51.0
Child	*n* = 2,088	*n* = 2,088	*n* = 8,865
Rural	91.3	80.0	60.6
Improved water source	88.3	87.2	95.6
Number of household members	6.1 (2.8)	6.1 (2.7)	7.2 (3.1)
Age (months)	32.4 (15.5)	32.7 (15.4)	24.9 (16.2)
Stunted	42.9	45.1	42.1
Wasted	10.8	10.4	16.0
Underweight	30.0	31.1	32.1
Anemic	46.4	46.0	62.5

a Reported as mean (SD) for continuous variables and percentage for dichotomous variables.

Estimates account for sampling weights.

WRA in Nepal were, on average (mean [SD]), 29.1 [9.8] years old, had given birth 2.0 [1.7] times, and had a BMI of 21.4 [3.4] kg/m^2^. WRA in Pakistan were on average (mean [SD]), 30.8 [6.2] years old, had given birth 3.9 [2.0] times, and had a BMI of 23.2 [5.1] kg/m^2^.

Anemia prevalence among children <5 years old in Nepal was 46.4% and 62.5% in Pakistan. In Nepal, 34.4% of WRA were anemic, whereas in Pakistan, the prevalence of anemia among WRA was 51.0%. Below, we present the results of factors associated with anemia among children <5 years old and WRA in Nepal, followed by children <5 years old and WRA in Pakistan.

### 3.1 Factors associated with anemia in Nepal

Factors associated with anemia among children <5 years old and WRA in Nepal are presented in Tables 2 and 3. Being a stunted child was associated with a higher prevalence of anemia compared with not being stunted (APR [95% CI]: 1.19 [1.08–1.30]), as was lower age. That is, the prevalence of anemia was greatest among infants <6 month old. At the same time, children with an anemic mother were more likely to be anemic compared to children of nonanemic mothers (1.31 [1.20–1.42]).

**Table 2:** Factors associated with anemia among children <5 years old in Nepal

	Initial model^a^		Final model^b^	
Variables	APR	(95% CI)	APR	(95% CI)
Environmental				
Month of interview				
Baisakh (April/May)	1.00		1.00	
Jestha (May/Jun)	0.86*	(0.74–1.01)	0.85**	(0.73–0.99)
Magh (January/February)	1.06	(0.86–1.31)	1.04	(0.86–1.27)
Falgun (February/March)	0.93	(0.79–1.11)	0.90	(0.76–1.06)
Chaitra (March/April)	0.87**	(0.76–1.00)	0.87**	(0.76–0.99)
Urban	1.00			
Rural	0.94	(0.81–1.08)		
Ecological zone				
Mountains	1.00		1.00	
Hills	0.89	(0.77–1.02)	0.87**	(0.76–1.00)
Terai	1.01	(0.85–1.20)	1.05	(0.89–1.23)
Household				
Wealth				
Richest	1.00			
Richer	1.11	(0.91–1.36)		
Middle	1.08	(0.88–1.32)		
Poorer	1.07	(0.87–1.31)		
Poorest	0.98	(0.78–1.24)		
Improved water	1.00			
Lack of improved water	1.11	(0.77–1.05)		
Facility at home	1.00		1.00	
Lack of facility at home	1.18***	(1.05–1.31)	1.22***	(1.12–1.33)
Female household head	1.00			
Male household head	1.00	(0.89–1.12)		
Smaller households^c^	1.00			
Larger households	1.05	(0.95–1.16)		
Hindu	1.00			
No Hindu	0.95	(0.82–1.09)		
Mother				
Age (years)				
15–24	1.00			
24–34	0.99	(0.89–1.09)		
35–49	0.99	(0.86–1.15)		
Body mass index (kg/m^2^)				
18.5–24.9	1.00			
<18.5	1.02	(0.92–1.14)		
≥25	0.98	(0.78–1.22)		
Education level				
No education	1.00			
Primary	0.95	(0.82–1.10)		
Secondary	0.94	(0.81–1.10)		
Higher	0.70**	(0.50–0.98)		
Literate	1.00			
Illiterate	0.96	(0.84–1.10)		
Not working	1.00			
Working	0.98	(0.90–1.08)		
Less births^c^	1.00			
More births	1.07	(0.94–1.21)		
Not anemic	1.00		1.00	
Anemic	1.30***	(1.19–1.42)	1.31***	(1.20–1.42)
Child				
Age (months)				
6–12	1.00		1.00	
13–24	0.80***	(0.73–0.88)	0.80***	(0.73–0.88)
25–36	0.55***	(0.48–0.63)	0.54***	(0.48–0.62)
37–48	0.44***	(0.38–0.51)	0.44***	(0.38–0.51)
49–59	0.29***	(0.24–0.35)	0.28***	(0.23–0.35)
Not stunted	1.00		1.00	
Stunted	1.14**	(1.03–1.28)	1.19***	(1.08–1.30)
Not wasted	1.00			
Wasted	1.02	(0.89–1.17)		
Not underweight	1.00			
Underweight	1.07	(0.96–1.20)		

*Note*. APR = adjusted prevalence ratio.

a Column represents adjusted prevalence ratios and 95% confidence intervals for a single modified Poisson model that included all variables.

b Column represents the remaining modified Poisson model after backwards stepwise regression (*p* < .05) was applied.

c Median split was used to dichotomize the variable.

* *P* < 0.05

** *P* < 0.01

*** *P* < 0.001.

**Table 3:** Factors associated with anemia among women in Nepal

	Initial model^a^		Final model^b^	
Variables	APR	(95% CI)	APR	(95% CI)
Environmental				
Month of interview				
Baisakh (April/May)	1.00		1.00	
Jestha (May/Jun)	0.87*	(0.73–1.02)	0.87*	(0.74–1.03)
Magh (January/February)	0.71***	(0.58–0.87)	0.70***	(0.58–0.85)
Falgun (February/March)	0.78***	(0.65–0.93)	0.78***	(0.67–0.92)
Chaitra (March/April)	0.83**	(0.71–0.98)	0.84**	(0.72–0.98)
Urban	1.00			
Rural	1.08	(0.92–1.26)		
Ecological zone				
Mountains	1.00		1.00	
Hills	1.09	(0.91–1.31)	1.08	(0.90–1.28)
Terai	1.93***	(1.59–2.34)	1.88***	(1.57–2.25)
Household				
Wealth				
Richest	1.00			
Richer	0.93	(0.81–1.06)		
Middle	0.98	(0.84–1.14)		
Poorer	1.03	(0.87–1.23)		
Poorest	1.01	(0.84–1.22)		
Improved water	1.00			
Lack of improved water	0.93	(0.77–1.14)		
Facility at home	1.00		1.00	
Lack of facility at home	1.08	(0.95–1.22)	1.10*	(0.98–1.21)
Female household head	1.00			
Male household head	1.08	(0.98–1.18)		
Smaller households^c^	1.00			
Larger households	0.96	(0.88–1.04)		
Hindu	1.00			
Not Hindu	0.96	(0.84–1.10)		
Individual				
Age (years)				
15–19	1.00		1.00	
20–24	0.92	(0.81–1.06)	0.93	(0.83–1.05)
25–29	0.89*	(0.77–1.02)	0.85***	(0.76–0.96)
30–34	0.90	(0.76–1.06)	0.86**	(0.76–0.98)
35–39	1.09	(0.91–1.30)	1.05	(0.92–1.19)
40–44	1.09	(0.91–1.31)	1.04	(0.92–1.18)
45–49	1.03	(0.83–1.27)	1.00	(0.85–1.16)
Body mass index (kg/m^2^)				
18.5–24.9	1.00		1.00	
<18.5	1.10*	(1.00–1.21)	1.10**	(1.00–1.21)
≥25	0.69***	(0.60–0.80)	0.70***	(0.61–0.81)
Education level				
No education	1.00			
Primary	0.93	(0.80–1.07)		
Secondary	1.01	(0.87–1.18)		
Higher	0.99	(0.79–1.22)		
Literate	1.00			
Illiterate	0.97	(0.86–1.10)		
Not working	1.00			
Working	1.01	(0.93–1.11)		
Less births^c^	1.00			
More births	0.97	(0.85–1.11)		
Not currently breastfeeding	1.00		1.00	
Currently breastfeeding	1.16***	(1.04–1.30)	1.17***	(1.07–1.28)
No amenorrhea	1.00			
Amenorrhea	1.11	(0.94–1.32)		

*Note*. APR = adjusted prevalence ratio.

a Column represents adjusted prevalence ratios and 95% confidence intervals for a single modified Poisson model that included all variables.

b Column represents remaining modified Poisson model after backwards stepwise regression (*p* < .05) was applied.

c Median split was used to dichotomize the variable.

* *P* < 0.05

** *P* < 0.01

*** *P* < 0.001.

Another risk factor for anemia among Nepalese children was living in a household without improved sanitation (1.22 [1.11–1.33]). Also, time of year of the interview and ecological zone of residence were also associated with anemia, in that children living in the hills were significantly less likely to be anemic than children in the mountains (reference region), but prevalence rates in the low‐lying Terai region did not differ from the mountains. Anemia was most prevalent among children in Baisakh (Nepali month of mid‐April to mid‐May) and significantly lower in Jestha (mid‐May to mid‐June) and Chaitra (mid‐March to Mid‐April).

Among WRA, being thin (BMI < 18.5 kg/m^2^) was associated with an increased likelihood of anemia (1.10 [1.00–1.21]), as was currently breastfeeding (1.17 [1.07–1.28]). Approximately 34% of women 15–19 years old were anemic, with significantly lower levels of anemia among women aged 25–29 and 30–34 years compared with women older than 34 years. WRA living in homes without any form of a sanitation facility (i.e., they defecate in bushes or in a field) were marginally more likely to be anemic than those with access to a sanitation facility (1.10 [0.98–1.21]). Woman's residence was also associated with anemia; approximately 40% of women in the Terai were anemic, whereas the prevalence of anemia in the hills and mountains was 25.1% and 24.7%, respectively. Month of interview was also associated with anemia among WRA in Nepal. Women assessed during Baisakh (mid‐April to mid‐May) were more likely to be anemic than was the month with the highest likelihood of anemia, with women assessed in any other months of the year less likely to be anemic.

### 3.2 Factors associated with anemia in Pakistan

Factors associated with anemia among children <5 years old and WRA in Pakistan are presented in Tables 4 and 5.

**Table 4:** Factors associated with anemia among children <5 years old in Pakistan

Variables	Initial model^a^		Final model^b^	
	APR	(95% CI)	APR	(95% CI)
Environmental				
Month of interview				
December to February	1.00			
March to May	1.04	(0.97–1.11)		
June to September	1.02	(0.91–1.13)		
Urban	1.00			
Rural	0.99	(0.94–1.03)		
Province				
Punjab	1.00			
Sindh	1.14***	(1.09–1.19)	1.14***	(1.09–1.18)
KPK	0.75***	(0.68–0.84)	0.77***	(0.70–0.85)
Balochistan	0.87*	(0.75–1.01)	0.87**	(0.76–0.99)
FATA	1.57***	(1.17–2.11)	1.74***	(1.47–2.05)
AJK	0.80***	(0.69–0.91)	0.79***	(0.71–0.88)
Gilgit	0.62***	(0.51–0.76)	0.64***	(0.54–0.76)
Household				
Wealth				
Richest	1.00		1.00	
Richer	1.03	(0.97–1.09)	1.03	(0.97–1.10)
Middle	1.04	(0.98–1.12)	1.05	(0.99–1.11)
Poorer	1.09**	(1.02–1.18)	1.09***	(1.03–1.16)
Poorest	1.09**	(1.01–1.19)	1.10***	(1.04–1.17)
Food security				
Food secure	1.00			
Mild food insecurity	1.01	(0.96–1.06)		
Moderate food insecurity	1.00	(0.95–1.06)		
Severe food insecurity	1.02	(0.96–1.10)		
No shared facility	1.00			
Shared facility	1.01	(0.97–1.05)		
Improved facility	1.00			
No improved facility	0.98	(0.92–1.04)		
Smaller households^c^	1.00			
Larger households	1.01	(0.97–1.05)		
Mother				
Age (years)				
15–24	1.00			
25–29	1	(0.95–1.06)		
30–24	1.02	(0.96–1.09)		
35–39	1	(0.93–1.08)		
40–49	1.02	(0.94–1.11)		
Body mass index (kg/m^2^)				
18.5–24.9	1.00		1.00	
<18.5	1.06**	(1.01–1.11)	1.05**	(1.01–1.10)
≥25	1.01	(0.96–1.05)	0.99	(0.95–1.03)
Literate	1.00			
Illiterate	1.01	(0.97–1.06)		
Less births^c^	1.00			
More births	0.97	(0.93–1.01)		
Number of children				
1	1.00			
2	1.01	(0.97–1.05)		
3 or more	1.01	(0.95–1.07)		
Not anemic	1.00		1.00	
Anemic	1.21***	(1.16–1.25)	1.21***	(1.17–1.26)
Child				
Took vitamin A supplement in past 6 months	1.00			
Did not take vitamin A supplement	1.00	(0.96–1.04)		
Not diagnosed with worms in past 6 months	1.00		1.00	
Diagnosed with worms	1.09*	(1.01–1.16)	1.06*	(1.00–1.13)
Took multiple micronutrients at some point	1.00			
Never took multiple micronutrients	1.02	(0.95–1.10)		
Age (months)				
<6	1.00		1.00	
6 through 11	1.18***	(1.09–1.28)	1.18***	(1.10–1.27)
12 through 23	1.26***	(1.17–1.35)	1.26***	(1.18–1.34)
24 through 35	1.15***	(1.06–1.24)	1.16***	(1.08–1.25)
36 through 59	0.86***	(0.79–0.94)	0.86***	(0.80–0.93)
Not stunted	1.00		1.00	
Stunted	1.10***	(1.05–1.14)	1.10***	(1.07–1.14)
Not wasted	1.00			
Wasted	1.01	(0.96–1.07)		
Not underweight	1.00			
Underweight	1.02	(0.98–1.07)		

*Note*. AJK = Azad Jammu and Kashmir; FATA = Federally Administered Tribal Area; KPK = Khyber Pakhtunkhwa; APR = adjusted prevalence ratio.

a Column represents adjusted prevalence ratios and 95% confidence intervals for a single modified Poisson model that included all variables.

b Column represents remaining modified Poisson model after backwards stepwise regression (*p* < .05) was applied.

c Median split was used to dichotomize the variable.

* *P* < 0.05

** *P* < 0.01

*** *P* < 0.001.

**Table 5:** Factors associated with anemia among women in Pakistan

	Initial model^a^		Final model^b^	
Variables	APR	(95% CI)	APR	(95% CI)
Environmental				
Month of interview				
December to February	1.00		1.00	
March to May	1.10**	(1.02–1.19)	1.10**	(1.02–1.18)
June to September	1.17***	(1.05–1.31)	1.17***	(1.05–1.29)
Urban	1.00			
Rural	0.98	(0.93–1.04)		
Province				
Punjab	1.00		1.00	
Sindh	1.20***	(1.14–1.27)	1.23***	(1.17–1.29)
KPK	0.77***	(0.68–0.88)	0.76***	(0.67–0.86)
Balochistan	0.89*	(0.77–1.02)	0.9	(0.79–1.03)
FATA	1.47*	(1.00–2.17)	1.06	(0.80–1.40)
AJK	0.73***	(0.62–0.86)	0.75***	(0.65–0.88)
Gilgit	0.51***	(0.41–0.64)	0.51***	(0.41–0.63)
Household				
Wealth				
Richest	1.00			
Richer	1.07	(0.98–1.16)		
Middle	1.07	(0.98–1.16)		
Poorer	1.05	(0.96–1.15)		
Poorest	1.09	(0.98–1.20)		
Food security				
Food secure	1.00			
Mild food insecurity	1.01	(0.95–1.08)		
Moderate food insecurity	1.03	(0.96–1.11)		
Severe food insecurity	1.07	(0.98–1.16)		
No shared facility	1.00			
Shared facility	0.98	(0.92–1.04)		
Improved facility	1.00			
No improved facility	1.04	(0.97–1.12)		
Smaller households^c^	1.00			
Larger households	1.03	(0.98–1.08)		
Individual				
Age (years)				
15–24	1.00			
25–29	1.03	(0.95–1.11)		
30–24	0.99	(0.91–1.08)		
35–39	1.02	(0.93–1.11)		
40–49	1.06	(0.96–1.17)		
Body mass index (kg/m^2^)				
18.5–24.9	1.00		1.00	
<18.5	1.07**	(1.01–1.13)	1.07**	(1.02–1.13)
≥25	0.83***	(0.78–0.88)	0.81***	(0.77–0.86)
Literate	1.00			
Illiterate	1.01	(0.96–1.07)		
Less births^c^	1.00		1.00	
More births	1.06*	(1.00–1.11)	1.09***	(1.04–1.14)
Took iron or folic acid during last pregnancy	1.00			
Did not take	0.98	(0.93–1.04)		
Took any supplements during last pregnancy	1.00		1.00	
Did not take	1.06**	(1.00–1.12)	1.09**	(1.04–1.14)
No symptoms	1.00			
Currently suffering from some symptoms	0.98	(0.94–1.03)		
Diet from past week				
Dairy consumption	1.00			
No dairy	1.01	(0.96–1.06)		
Meat consumption	1.00			
No meat	0.98	(0.91–1.05)		
Eggs consumed	1.00			
No eggs	1.02	(0.97–1.07)		

*Note*. AJK = Azad Jammu and Kashmir; FATA = Federally Administered Tribal Area; KPK = Khyber Pakhtunkhwa; APR = adjusted prevalence ratio.

a Column represents adjusted prevalence ratios and 95% confidence intervals for a single modified Poisson model that included all variables.

b Column represents remaining modified Poisson model after backwards stepwise regression (*p* < .05) was applied.

c Median split was used to dichotomize the variable.

* *P* < 0.05

** *P* < 0.01

*** *P* < 0.001.

Age was associated with anemia among children; children aged 6–11 and 12–23 months were more likely to be anemic than children aged <6 months, whereas children 36–59 months old were less likely to be anemic than children <6 months old. Stunted children were more likely to be anemic than nonstunted children (1.10 [1.07–1.14]), and having been diagnosed with worms in the past 6 months also increased a child's likelihood of anemia (1.06 [1.00–1.13]) as well. Children with an anemic mother were more likely to be anemic than children with a nonanemic mother (1.21 [1.17–1.26]), as were children with thin mothers compared to children with normal weight mothers (1.05 [1.01–1.10]). Children from the lower two wealth quintiles were more likely to be anemic compared with children from the richest wealth quintile (poorer: 1.09 [1.03–1.16] and poorest: 1.10 [1.04–1.17]). Province of residence was also associated with anemia.

Examining the association between micronutrient deficiencies (vitamin A, vitamin D, iron, and zinc) and anemia among children in bivariate models adjusted for the survey design revealed that children with iron depletion were more likely to be anemic than children who were not iron deplete (1.42 [1.04–1.47]).

Among WRA, thinness (1.07 [1.02–1.13]) and having given birth more than the median number of times in this population (two births; 1.09 [1.04–1.14]) were both risk factors of anemia, as was not taking any supplements during pregnancy (1.09 [1.04–1.14]; Table 5). The prevalence of anemia ranged across provinces and season. Compared with women interviewed between December and February, WRA interviewed in March through May and June through September were more likely to have higher likelihood of being anemic (1.10 [1.02, 1.18], 1.17 [1.05–1.29], and 1.20 [1.08, 1.33], respectively).

We also examined the association between micronutrient deficiencies (vitamin A, vitamin D, calcium, iron, and zinc) and anemia among WRA in bivariate models adjusted for the survey design. We found that women with iron depletion (1.56 [1.50–1.63]) and also vitamin A deficiency (1.11 [1.06–1.15]) were more likely to be anemic than women who were not iron deplete or vitamin A deficient.

## 4 DISCUSSION

On the basis of the samples used in this analysis, the prevalence of anemia among children in Nepal was 46%, whereas more than one third of WRA were also affected. In Pakistan, the prevalence among children and WRA was 63% and 51%, respectively. Below, we consider the findings from this study within the context of recent work conducted in South Asia, such as the recent studies from Bangladesh and India described earlier.

### 4.1 Anemia among children <5 years old

Age of child and being stunted were associated with a higher risk of anemia in children for both Nepal and Pakistan. This is consistent with a recent nationally representative study conducted in Bangladesh (Khan, Awan, & Misu, 2016). In Nepal, the prevalence of anemia was greatest among infants aged 0–5 months. Similarly, in Bangladesh, children aged 6–23 months carried a higher risk for anemia compared with children 24–59 months old (Khan et al., 2016). In Pakistan, children aged 12–23 months had the highest prevalence of anemia (72.5%) compared with children 36–59 months old (50.4%). This is consistent with a study conducted among 12 to 23 month old children in two rural districts of India, which showed that child's age was positively associated with hemoglobin concentration (Pasricha et al., 2010).

This is an important finding in the context of 1,000 Days programing, which has become the focus of many governments and donors. The period of conception up to a child's second birthday is critical both to maternal health and nutrition, to healthy birth outcomes, and to subsequent child growth and development. It is therefore important to recognize that in South Asia, when mothers are anemic, infants are often born anemic. This suggests a need to focus on much policy attention aimed at resolving anemia to pregnant and lactating mothers, as well as to infants and young children in the context of introducing appropriate iron‐rich complementary foods at 6 months of age as well as optimizing other infant and young child feeding practices.

It also suggests that tackling anemia should be a focus of broader initiatives aimed at improving child growth. The international community and many national governments have made a reduction in child stunting (another World Health Assembly target for 2025) a policy priority. Advocacy for this is strong, including by the influential *Scaling up Nutrition* movement and the *Stop Stunting* initiative that is focused on South Asia. Stunted children in Bangladesh have been shown to have a higher likelihood of being anemic than nonstunted children, but this was not observed in India (Baranwal, Baranwal, & Roy, 2014; Khan et al., 2016). This means that careful analysis has to underpin cost‐effective programing and that targeted actions with tailored activities are likely to gain more traction than conventional one‐size‐fits‐all investments. Simply promoting child growth (while needed) may not in itself reduce anemia, and vice versa.

For example, studies have been inconsistent with regard to the effect of iron supplementation on linear growth. One systematic review concluded that iron supplementation provides no apparent benefit to linear growth among children (Ramakrishnan, Nguyen, & Martorell, 2009). However, that systematic review did not account for the difference in supplementing iron‐deplete versus iron‐replete children. As a result, although iron supplementation did not in their assessment appear to impact growth, it is likely that treating anemia among specifically anemic children could contribute to improving linear growth. At the same time, a reduction in stunting could help in managing anemia if appropriate actions were taken. Given the overlap of anemia and stunting among children across South Asia, there is an opportunity to target both conditions simultaneously.

Not having improved sanitation in the home and poorer wealth index were determinants of anemia in Nepal and Pakistan, respectively. Similarly, nonimproved toilet facilities and water sources as well as poorer wealth index were all determinants of anemia in Bangladesh (Khan et al., 2016). Extensive research has demonstrated the link between indicators of socioeconomic status and nutrition (Adler & Ostrove, 1999; Adler et al., 1994), and it is well established that socioeconomics is an important determinant of anemia (Balarajan et al., 2011); thus, our findings reinforce the need for targeted efforts to focus on children from poor, hard to reach, and socially excluded households and population groups.

### 4.2 Anemia among WRA

In our analysis, BMI was inversely associated with anemia in WRA in Nepal and Pakistan, which is in line with findings from India and Bangladesh (Balarajan et al., 2013; Kamruzzaman et al., 2015). Overweight and obese women had a lower prevalence of anemia compared with women of normal weight. However, the prevalence of anemia in overweight women was 20–22% in Nepal and 46–47% in Pakistan, indicating that the double burden of malnutrition (overweight or obesity alongside underweight and nutritional deficiencies) is already present in these countries and needs serious policy attention.

In Pakistan, the likelihood of anemia was greater among women who had given birth more than twice, whereas in India, having five or more children was associated with a higher prevalence of anemia compared with having no children (Balarajan et al., 2013). Thus, the physiological burden of repeated pregnancy, likely coupled with low‐quality diets, parasites, and poor sanitation, poses important risks to mother and their babies.

Women's years of schooling was not a determinant of anemia in Nepal or Pakistan. That contrasts with Bangladesh and India, where an inverse relationship was found between years of formal education and anemia (Kamruzzaman et al., 2015) (Balarajan et al., 2013). In a pooled analysis across low‐income countries, education level along with wealth and cultural norms and behaviors were overarching determinants of anemia among women (Balarajan et al., 2011). The lack of association in our analysis for Nepal and Pakistan suggests that factors that are relatively less amenable to the influence of education (such as low quality of diet, parasite loads, and lack of access to clean water and sanitation facilities) may be playing a more dominant role in anemia.

Interestingly, the same contrasts hold in relation to household wealth between Nepal and Pakistan on one hand (where wealth index was not correlated with anemia) and India and Bangladesh on the other hand (where low wealth was a factor in anemia prevalence). Similarly, religion was not associated with anemia in Nepal, although it was in India and Bangladesh. In Bangladesh, which is a predominately Muslim country, non‐Muslim women have a higher prevalence of anemia compared with Muslims (49.4% vs. 40.2%; Kamruzzaman et al., 2015). In India, a predominately Hindu country, Muslims (55.7%) and Christians (51.2%) had a slightly lower risk of anemia compared with Hindus (56.6%; Balarajan et al., 2013).

### 4.3 Policy implications

Iron deficiency anemia is one of the top 10 leading causes of YLD in South Asia (and within the top 5 in Afghanistan, Bangladesh, India, and Nepal, 2013), with notable human and economic costs to countries. The median cost of iron deficiency for 10 developing countries was found to be $2.32 per capita (0.57% of GDP) based on annual physical productivity losses alone (Horton & Ross, 2003). When both physical and cognitive productivity losses were accounted for, this increased to $16.78 per capita (over 4% of GDP).

Programs that target specific known forms of anemia can be cost‐effective. In calculating the cost‐effectiveness of iron supplementation and fortification, Baltussen found that iron supplementation would avert ~2.5 million disability‐adjusted life years (DALYs) in Africa and Southeast Asia at 95% coverage (Baltussen, Knai, & Sharan, 2004). In Southeast Asia, the cost of iron supplementation at 95% coverage would be 115 international dollars per DALY averted. For iron fortification, the cost would be 35 international dollars per DALY averted.

In addition to supplementation and fortification, efforts to increase dietary intake of iron‐rich foods across South Asia are needed. Availability and geographical and financial access may be barriers to these dietary changes, as well as religious and cultural beliefs. Homestead food production programs have shown to change diets and reduce anemia among women and children. This is a promising intervention that has been used in Bangladesh and Nepal, among other countries (Olney, Pedehombga, Ruel, & Dillon, 2015; Talukder et al., 2010).

Although fortification and supplementation are cost‐effective approaches to preventing and treating anemia due to nutritional deficiencies, other potential causes of anemia cannot be overlooked. Certain infectious diseases and genetic disorders can also cause anemia. In such cases, micronutrient supplementation or fortification will not correct anemia (Balarajan et al., 2011). Water, hygiene and sanitation interventions, deworming, and malaria prevention and treatment are interventions that may be considered alongside dietary improvement and micronutrient supplementation and fortification, depending on the context‐specific conditions. Genetic hemoglobin disorders in developing countries pose an added barrier given the low diagnostic capabilities for these disorders in remote and resource poor areas.

The policy commitment and scope to combat anemia in children and women in South Asian countries vary. In response to a 75% prevalence of anemia among pregnant women in 1998, the government of Nepal developed the *National Strategy for the Control of Anemia among Women and Children in Nepal* in 2003 and formulated the *Intensification of Maternal and Neonatal Micronutrient Program* (*IMNMP*) with technical support by UNICEF and the micronutrient initiative (Pokharel, Maharjan, Mathema, & Harvey, 2011). The program began as an operational research in August 2003. After finding that bringing services closer to communities through Female Community Health Volunteers increased iron and folic acid (IFA) coverage and compliance, the program was gradually scaled up to 74 of the 75 districts between 2003 and 2012. That program targeted antenatal care (ANC) visits, IFA supplementation, and deworming prophylaxis during pregnancy and utilized the existing infrastructure of the Female Community Health Volunteers. Between 1998 and 2006, anemia among women dropped from 68% to 36%, a reduction that was attributed to this program.

However, since 2006, the prevalence of anemia only improved by 1% by 2011 despite the continued efforts of the IMNMP. Because anemia is caused by many factors, apart from iron deficiency and worm infestation, the IMNMP program could contribute to the reduction of anemia due to iron deficiency and worm infestation, but other causal factors will need to be explored. The success of Nepal in reducing anemia between 1998 and 2006 is encouraging. It is expected that the Nepal National Micronutrient Status Survey 2016 (findings expected in 2017) will document the multifactorial etiology of anemia in Nepal and provide an evidence base to revise the national strategy for the control of anemia that can bring about further reductions on anemia among children, adolescents, and women.

The Ministry of Health of Pakistan developed a *Maternal and Child Health Policy and Strategic Framework* (2005–2015) that included little focus on anemia prevention, though the framework did include nutrition advice and micronutrient supplementation. A large‐scale fortification program is underway, including the fortification of wheat flour with iron, which is expected to reduce the prevalence of anemia. Although the fortification program is promising, Pakistan may need a more comprehensive strategy to reduce anemia—particularly among young children, who are likely to benefit less from large‐scale fortification of wheat flour with iron—that considers the multifactorial etiology of anemia, as seen in countries such as Bangladesh and Nepal and the potential for impact of each of the interventions in the population groups at a higher risk of anemia (i.e., young children, adolescents, and WRA).

## 5 CONCLUSIONS

Bangladesh is the only country in South Asia that is on track to meet the World Health Assembly goal to reduce by 50% the prevalence of anemia in WRA by 2025. This argues for much greater policy attention and programatic investment to tackle anemia across the South Asia region. Progress in Nepal was rapid between 1998 and 2006, falling from 65% to 34%, but since 2007 has stalled (World Health Organization, 2014). It is widely believed that iron supplementation activities helped secure the early progress but that other determinants are in need of being addressed. Efforts are currently underway in Nepal to determine how best to address the multifactorial etiology of anemia.

In Pakistan, as in neighboring Afghanistan and India, the prevalence of anemia appears to have increased over the past decade or so. Much more needs to be understood about the drivers of such regression. The achievement of the global target of cutting anemia by half among WRA requires a relative rate of reduction exceeding 5% per year. The target uses the 1993–2005 period as its reference point for the 50% reduction, which underlines the fact that many parts of South Asia have a huge task ahead of them—first managing to reverse recent trends and then accelerating reduction at rates that will need to reach almost 10% relative reduction per year by 2025 (World Health Organization, 2014).

With millions of anemic women and children in South Asia, governments of this region have to take urgent action. Part of this will involve tackling endemic malaria, but actions are needed across multiple sectors in coordinated ways. Targeting higher risk groups with well‐implemented evidence‐based interventions that combine improvements in diets, micronutrient supplementation, and food fortification to address nutritional anemia while responding to the multifactorial etiology of anemia will utilize resources more effectively and yield larger impacts. Continued monitoring of risk factors for anemia and progress in program implementation will inform policymakers, program implementers, and researchers of changing and stagnant patterns across time and geographies.

## CONFLICT OF INTEREST

The authors declare that they have no conflicts of interest.

## AUTHORS CONTRIBUTIONS

KLH, PW, GN, and VMA designed the research. KLH wrote the first draft of the paper, and all authors edited and approved the final paper.
